# CRISPR/Cas9-Mediated Mutagenesis of *Carotenoid Cleavage Dioxygenase 8* (*CCD8*) in Tobacco Affects Shoot and Root Architecture

**DOI:** 10.3390/ijms19041062

**Published:** 2018-04-02

**Authors:** Junping Gao, Tong Zhang, Bingxin Xu, Ling Jia, Bingguang Xiao, He Liu, Lijing Liu, Hao Yan, Qingyou Xia

**Affiliations:** 1 State Key Laboratory of Silkworm Genome Biology, Southwest University, Chongqing 400715, China; gaojp@swu.edu.cn (J.G.); tong2016@email.swu.edu.cn (T.Z.); x7251012@email.swu.edu.cn (B.X.); jialing132@163.com (L.J.); lh694215870@email.swu.edu.cn (H.L.); liulijing1@email.swu.edu.cn (L.L.); yanhao@email.swu.edu.cn (H.Y.); 2 Key Laboratory of Tobacco Biotechnological Breeding, National Tobacco Genetic Engineering Research Center, Yunnan Academy of Tobacco Agricultural Sciences, Kunming 650021, China; xiaobg@cqu.edu.cn

**Keywords:** *carotenoid cleavage dioxygenase* (*CCD*), *Nicotiana tabacum*, plant architecture, shoot branch-ing, Strigolactones, targeted mutagenesis

## Abstract

Strigolactones (SLs) are a class of phytohormones that regulate plant architecture. *Carotenoid cleavage dioxygenase* (*CCD*) genes are involved in the biosynthesis of SLs and are identified and characterized in many plants. However, the function of *CCD* genes in tobacco remains poorly understood. In this study, two closely related genes *NtCCD8A* and *NtCCD8B* were cloned from tobacco (*Nicotiana tabacum* L.). The two *NtCCD8* genes are orthologues of the tomato (*Solanum lycopersicum*) *carotenoid cleavage dioxygenase* 8 (*SlCCD8*) gene. *NtCCD8A* and *NtCCD8B* were primarily expressed in tobacco roots, but low expression levels of these genes were detected in all plant tissues, and their transcript levels significantly increased in response to phosphate limitation. *NtCCD8A* and *NtCCD8B* mutations were introduced into tobacco using the CRISPR/Cas9 system and transgenic tobacco lines for both *ntccd8* mutant alleles were identified. The *ntccd8a* and *ntccd8b* mutant alleles were inactivated by a deletion of three nucleotides and insertion of one nucleotide, respectively, both of which led to the production of premature stop codons. The *ntccd8* mutants had increased shoot branching, reduced plant height, increased number of leaves and nodes, and reduced total plant biomass compared to wild-type plants; however, the root-to-shoot ratio was unchanged. In addition, mutant lines had shorter primary roots and more of lateral roots than wild type. These results suggest that *NtCCD8* genes are important for changes in tobacco plant architecture.

## 1. Introduction

Strigolactones (SLs) are new types of plant hormones that regulate many aspects of plant growth and developmental processes. Strigol was the first characterized SL, which was exuded from cotton roots and induces the germination stimulant of *Striga lutea* [1]. SLs are mainly produced in plant roots, and many SLs have been identified in root exudates and root extracts from different plant species [2,3,4]. SLs have also been identified as rhizosphere signaling molecules in arbuscular mycorrhizal (AM) symbiosis, which improve the ability of the uptake of nutrients in plants [5,6,7]. Consistent with their role in nutrient uptake, SLs synthesis and release increase under phosphate limitation [8,9,10].

As a newly discovered endogenous hormones, SLs play a role in controlling above- and below-ground plant architecture [11,12,13,14,15]. As an important component of plant architecture, shoot branching is determined by SLs [16,17]. Several SL-pathway mutants have elevated shoot-branching, such as ramosus (*rms*) in pea (*Pisum sativum*), more axillary growth (*max*) in *Arabidopsis*, decreased apical dominance (*dad*) in petunia (*Petunia hybrida*), and *dwarf* (*d*) or high-tillering dwarf (*htd*) in rice (*Oryza sativa*) [18,19,20,21,22]. Besides functioning as phytohormones that control shoot-branching patterns, SLs have been shown to regulate plant root growth and development, promote lateral and adventitious root formation, and reduce primary root and root hair length in SL-deficient and SL-insensitive mutants [23,24,25]. SLs also contribute to the regulation of secondary growth, leaf shape, leaf senescence and in drought and salinity responses [26,27,28,29,30,31]. However, SLs regulate plant growth and development in conjunction with other plant hormones, such as auxin, abscisic acid (ABA), cytokinins (CK), ethylene, jasmonic acid (JA), gibberellin (GA) and brassinosteroids (BR) [28,31,32,33,34,35,36,37].

Strigolactones are a group of carotenoid-derived molecules [9,38]. More genes have been recently identified and demonstrated to be involved in the biosynthesis of SLs through the study of SL-deficient mutants [11,39,40]. The all-trans-β-carotene is first converted into 9-cis-β-carotene by an iron-containing D27 enzyme [41]. The 9-cis-β-carotene substrate is then sequentially cleaved by carotenoid cleavage dioxygenase 7 (CCD7) and carotenoid cleavage dioxygenase 8 (CCD8). CCD7 cleaves the 9′,10′ double bond in 9-cis-β-carotene to give rise to 10′-apo-β-carotenal and β-ionone [19,42,43]. The 10′-apo-β-carotenal is then cleaved by CCD8 to produce carlactone, which is a precursor of SLs [41]. *CCD7* and *CCD8* genes have been identified in the SLs synthetic pathways of several plant species, including *Arabidopsis*, pea, rice, tomato, saffron, *Phelipanche aegyptiaca*, maize, apple, poplar and petunia [19,43,44,45,46,47,48,49,50,51,52,53,54]. The expression levels of *SlCCD8* and *SlCCD7* down-regulated by RNA interference reduced strigolactone concentrations in tomato [43,46]. Orthologs of *CCD8* were also characterized in potato, maize, chrysanthemum and kiwifruit [55,56,57,58]. The carlactone is then catalyzed by the cytochrome P450 more axillary growth 1 (MAX1) protein to produce carlactonoic acid (CLA) [59]. MAX1 has been shown to participate in the SLs biosynthesis pathway and has been characterized in *Arabidopsis*, pea, petunia and rice [20,50,60,61,62]. CLA is then methylated to give rise to methyl carlactonate (MeCLA) by an unknown enzyme, and subsequently transformed into an unknown compound by lateral branching oxidoreductase (LBO) [40].

Tobacco (*Nicotiana tabacum* L.) is widely cultivated worldwide as a non-food crops, and is also an important model plant species for basic biological research. To date, the genes involved in the SLs signaling system have been identified in *Arabidopsis*, pea and rice [39,63,64], however, no genes in the SLs biosynthetic pathway have been reported in tobacco. Understanding the SLs biosynthetic pathway in tobacco would contribute to describing the functional characterization of SLs involved in tobacco growth and development. In recent years, genome-editing tools including zinc finger nucleases (ZFNs), transcription activator-like effector nucleases (TALENs) and the clustered regularly interspaced short palindromic repeats (CRISPR)-associated protein 9 (Cas9) system (the CRISPR/Cas9 system) have been used in the genome modification of tobacco [65,66,67,68]. In this study, two *NtCCD8* genes from tobacco were cloned and their role in SLs biosynthesis and tobacco plant growth and development was characterized via the targeted knockout using the CRISPR/Cas9 system.

## 2. Results

### 2.1. Identification of NtCCD8A and NtCCD8B Genes in Tobacco

**Figure 1:**
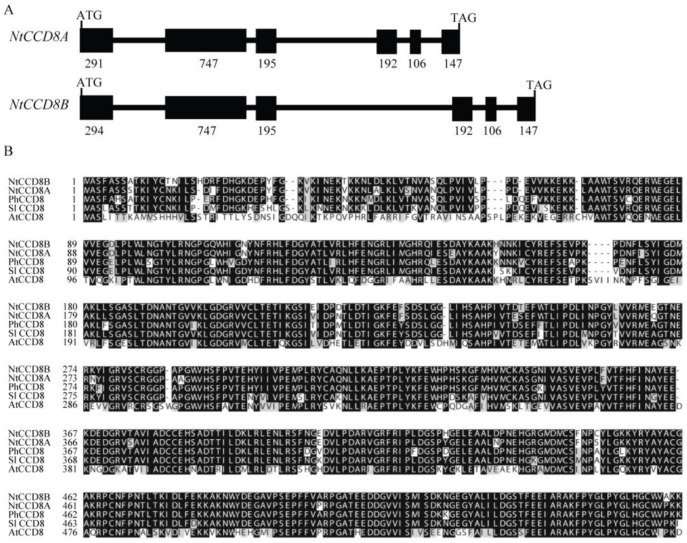

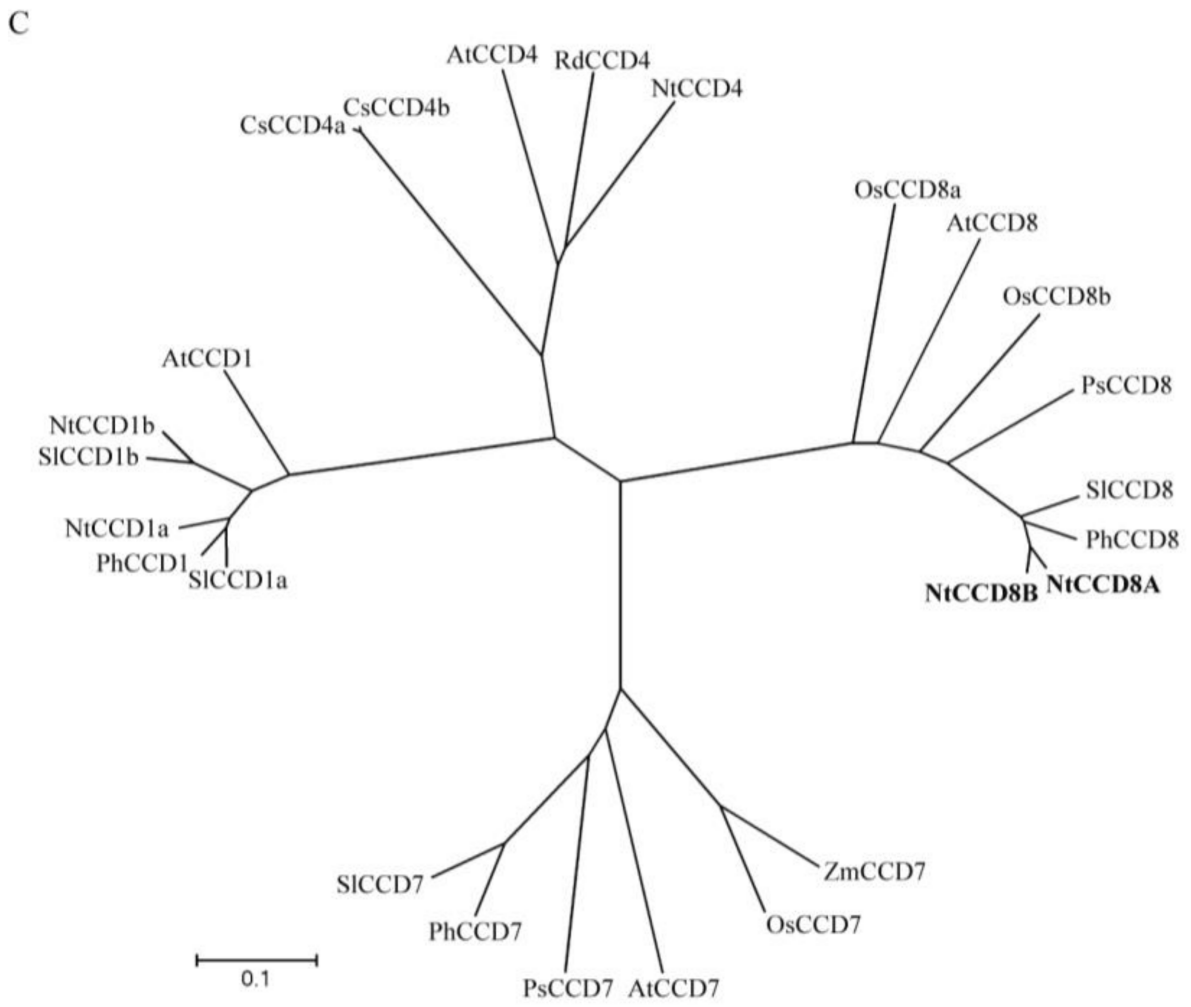
Characterization of *NtCCD8* genes in tobacco (*Nicotiana tabacum*). (**A**) Putative exon/intron structure of the *NtCCD8A* and *NtCCD8B* genes. (**B**) Alignment of the putative NtCCD8A and NtCCD8B amino acid sequences with known CCD8 proteins from other plant species. Nt, *Nicotiana tabacum*; At, *Arabidopsis thaliana*. Ph, *Petunia hybrida*; Sl, *Solanum lycopersicum*. (**C**) Phylogenetic tree obtained for known CCD1, CCD4, CCD7 and CCD8 amino acid sequences; Nt, *Nicotiana tabacum*; At, *Arabidopsis thaliana*; Ph, *Petunia hybrida*; Sl, *Solanum lycopersicum*. Os, *Oryza sativa*; Ps, *Pisum sativum*; Zm, *Zea mays*; Cs, *Crocus sativus*; Rd, *Rosa damascena*. Accession numbers for the sequences used are as follows: AtCCD1 (At3g63520), SlCCD1a (AAT68187), SlCCD1b (AAT68188), NtCCD1a (AIL30506), NtCCD1b (AKO22630), CsCCD4a (ACD62476), CsCCD4b (ACD62477), RdCCD4 (ABY60886), NtCCD4 (AEI61930), OsCCD7 (Q7XU29), ZmCCD7 (NP_001183928), AtCCD7 (AEC10494), PsCCD7 (ABD67496), PhCCD7 (FJ790878), SlCCD7 (ACY39883), OsCCD8a (AP003296), OsCCD8b (AP003376), AtCCD8 (AEE86121), PsCCD8 (AY557341), SlCCD8 (AEH96363), and PhCCD8 (AY743219).

In order to identify the tobacco *CCD8* gene homologue, amino acid sequences of SlCCD8 and PhCCD8 as the query were aligned against the Sol Genomics Network (SGN) tobacco genome database (http://solgenomics.net/organism/Nicotiana_tabacum/genome) using a tBLASTn search. Two pu-tative coding sequences (mRNA_112966_cds and mRNA_104099_cds) from tobacco *CCD8* genes were identified from the tBLASTn search against the SGN database. These nucleotide sequences were used to design the specific primers for the full-length CCD8 coding sequences, which were amplified from tobacco root tissues. The tobacco *CCD8* genes have two open reading frames (ORF) that are 1668 bp and 1671 bp in length and encode 556 and 557 amino acid proteins, respectively. Based on their high identity to SlCCD8, tobacco CCD8 proteins were named NtCCD8A and NtCCD8B. The nucleotide sequences of both *NtCCD8* genes from the tobacco genome database were aligned with the ORF sequences, and the two *NtCCD8* genes were predicted to have six exons (Figure 1A). Alignment of NtCCD8A with NtCCD8B, SlCCD8, PhCCD8 and AtCCD8 showed 96% amino acid sequence identity with NtCCD8B, moreover, the tobacco CCD8 proteins, were 88% identical to SlCCD8, 90–92% identical to PhCCD8, and 68–76% identical to AtCCD8 (Figure 1B and Table S1). The Neighbor-Joining (NJ) phylogenetic tree constructed for CCD8 proteins from several plants showed a clear evolutionary separation between NtCCD8 and its homologues, except for PhCCD8 and SlCCD8 with which they were grouped (Figure 1C).

### 2.2. Expression Patterns of NtCCD8 Genes in Different Tobacco Tissues and Stress Conditions

**Figure 2:**
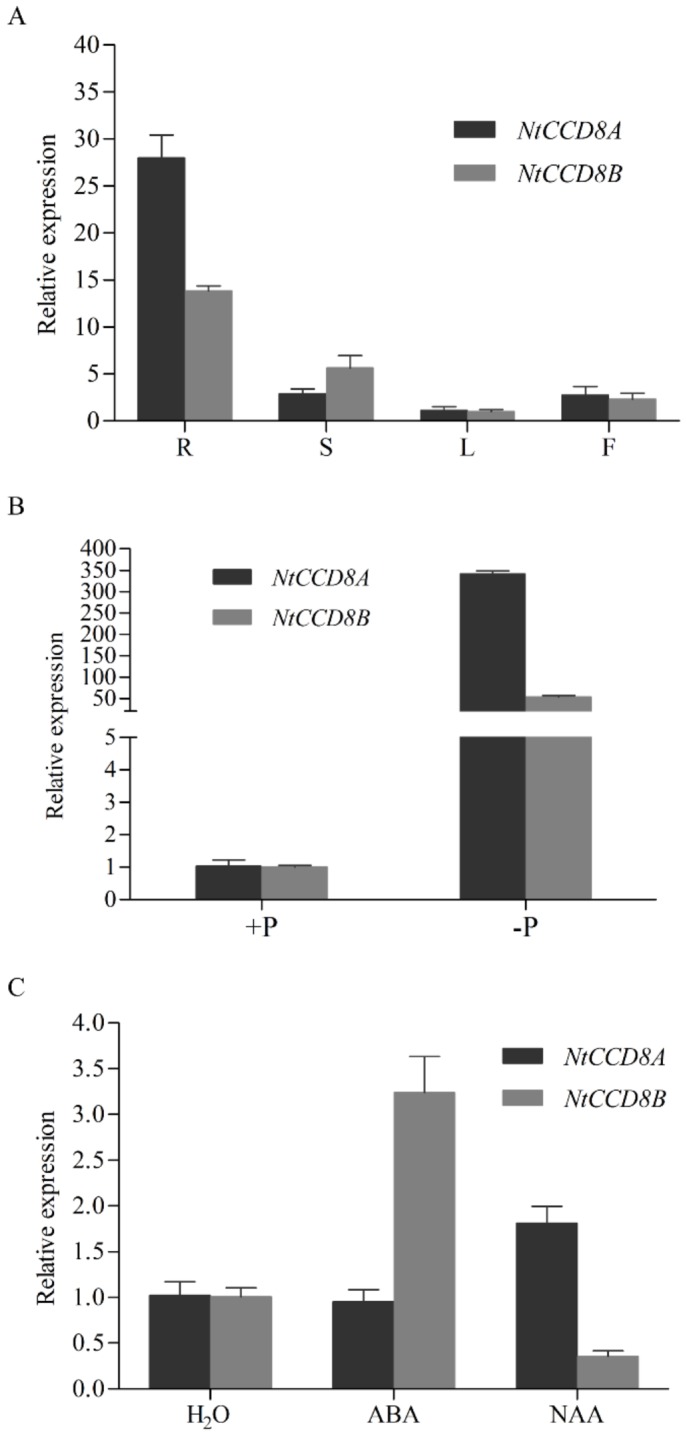
*NtCCD8A* and *NtCCD8B* expression patterns in several tissues and in response to stress conditions. (**A**) Relative gene expression of *NtCCD8A* and *NtCCD8B* in the several tissues of wild-type tobacco plants: R, root; S, stem; L, leaf; F, flower. (**B**) qRT-PCR analysis of *NtCCD8A* and *NtCCD8B* transcript levels in the root tissues of wild-type tobacco plants: in the presence or absence of phosphate (P). (**C**) qRT-PCR analysis of *NtCCD8A* and *NtCCD8B* transcript levels in young seedlings treated with 100 μM ABA and 100 μM NAA for 6 h. Data are presented as means ± SD. Error bars represent standard deviation of three replicates.

To examine the expression level of *NtCCD8* genes in wild-type tobacco plants, qRT-PCR was used to analyze RNA transcript levels in different tissues. *NtCCD8A* and *NtCCD8B* expression were detected in all plant tissues but was most abundant in roots. Notable, the difference in expression level between roots and leaves is lower for *NtCCD8B* than for *NtCCD8A* (Figure 2A). 

In order to investigate the *NtCCD8* expression pattern in response to various stress, tobacco seedlings were treated with ABA, 1-Naphthaleneacetic acid (NAA) and nutrient deficiency. *NtCCD8A* and *NtCCD8B* expression in root tissues increased under phosphate starvation, and *NtCCD8A* expression was six-fold higher than that of *NtCCD8B* (Figure 2B). NAA treatment induced expression of *NtCCD8A* and repressed that of *NtCCD8B* (Figure 2C). ABA treatment increased the expression level of *NtCCD8B*, but not of *NtCCD8A* (Figure 2C).

### 2.3. Targeted NtCCD8 Mutations Using the CRISPR/Cas9 System

**Figure 3:**
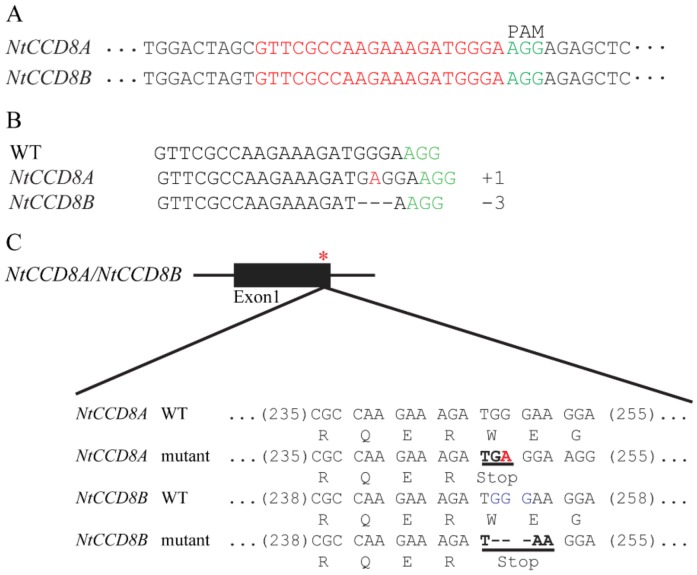
CRISPR/gRNA-mediated *NtCCD8* genes disruption. (**A**) The common target site selected for targeted *NtCCD8A* and *NtCCD8B* mutation. The target region is indicated by the nucleotides in red followed by PAM (proto-spacer adjacent motif). (**B**) Sequences of the mutated sites in homozygous *ntccd8* mutant plants. Deletions and insertions are indicated by dashes and red letters, and the numbers on the right side indicate the size of the deletions and insertions. (**C**) Schematic representation of the mutated position in *ntccd8* mutant plants. The position of premature stop codons caused by deletions and insertions is indicated by a red asterisk, the deleted “GGG” and inserted “A” bases are shown in blue and red lettering, respectively, and premature stop codons are shown in bold.

To investigate the biological function of *NtCCD8* genes in tobacco, *ntccd8* mutant plants were produced using the CRISPR/Cas9 system. The common target site (20 nucleotides) in *NtCCD8A* and *NtCCD8B* was selected and this sequence is located adjacent to protospacer adjacent motif (PAM), which is essential for Cas9 to recognize and cleave the target site (Figure 3A). The 20 bp target sequence was introduced into a binary expression vector, that was then transformed into tobacco with *Agrobacterium tumefaciens*-mediated transformation. Eighteen T_0_ transgenic lines were obtained and evaluated for mutations. Mutations were identified in 14 of the 18 transgenic plants with the percentage of 77.8% (Table 1). Meanwhile, three of the eighteen T_0_ transgenic plants had more shoot branching than wild-type tobacco plants, and the mutations in the target site were detected with Sanger sequencing (Figure S1). The genotypic analysis of T_1_ plants showed that the *NtCCD8* mutations introduced into T_0_ lines were inherited and the transgenic region had been eliminated through self-cross in some plants (Table 2). In homozygous T_2_ mutant plants, a one-base A insertion and three-base GGG deletion were detected in the first exon of *NtCCD8A* and *NtCCD8B*, respectively (Figure 3B). The insertion was located at position 249 in *NtCCD8A* and the deletion was located at position 251 in *NtCCD8B*, both of which produced premature stop codons (TAA and TGA) in the ORF and created loss-of-function mutants (Figure 3C). The one most likely off-target site was selected and examined in T_2_ mutant plants (Table S2). Results showed that no mutations were observed in the selected candidate off-target site.

### 2.4. Targeted NtCCD8 Mutations Affect Shoot Architecture

**Figure 4:**
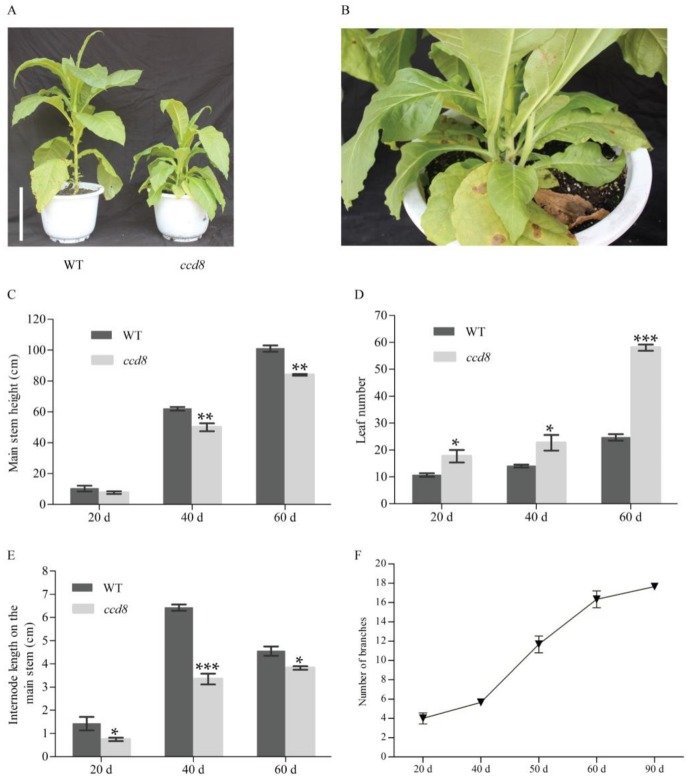
Shoot phenotype in *ntccd8* mutant and wild-type plants. (**A**) Photograph of 40-day-old wild-type and *ntccd8* mutant plants. The scale bars equal to 20 cm. (**B**) Detail of primary branches in 40-day-old *ntccd8* mutant plants. Main stem length (**C**), leaf number (**D**) and internode length of the main stem (**E**) were determined at 20 d, 40 d and 60 d after transplanting, respectively. (**F**) Total number shoot branches in *ntccd8* mutant plants at 20 d, 40 d, 50 d, 60 d and 90 d after transplanting. Data are represented as the means ± SD (*n* = 10 for wild-type plants versus *n* = 12 for mutant plants). The asterisks indicate statistically significant differences compared to wild-type plants (* *p* < 0.05, ** *p* < 0.01 and *** *p* < 0.001; Student’s *t*-test).

SLs was analyzed in wild-type tobacco and *ntccd8* mutant plants using a ultra-performance liquid chromatography tandem spectrometry (UPLC-MS/MS) method. 5-Deoxystrigol, one of SLs, was detected in the root extracts of wild-type plants, but it not in the root extracts of *ntccd8* mutants (Figure S2). The morphology of *ntccd8* mutant lines was characterized and evaluated. The mutant lines showed obviously different phenotype compared to wild-type plants, which were remarkably more branched (Figure 4A,B). At 20 days after transplant (dat), the axillary buds of mutant lines were visualized, growing into numerous lateral stems as observed at 40 dat and 90 dat. (Figure 4A and Figure S7). Mutant plant height, stem perimeter and internode length are were lower than those of wild-type plants, whereas leaf and node number, branch length and number were higher (Figure 4B–F and Figure S3). However, mutant and wild-type plants’ height was not significantly different at the early development stage (Figure 4C). The number of leaves in *ntccd8* mutant plants increased markedly between 40 and 60 dat (Figure 4D). Although node number at 20 and 40 dat was higher in mutants than in wild-type plants, this number remained unchanged at 60 dat; at this time internode length was shorter in *ntccd8* mutants than in wild-type plants, due to mutants being shorter plant height (Figure 4E and Figure S3A). All *ntccd8* mutant plants displayed a 30% reduction of main stem perimeter compared to wild-type plants (Figure S3B). In addition, the mutant plants had a rapidly increase branch number in the period between 40 and 60 dat, whereas the length of the highest lateral branch was increased rapidly in the period between 60 to 90 dat (Figure 4F and Figure S3C).

### 2.5. Targeted NtCCD8 Mutations Affect Root Morphology

**Figure 5:**
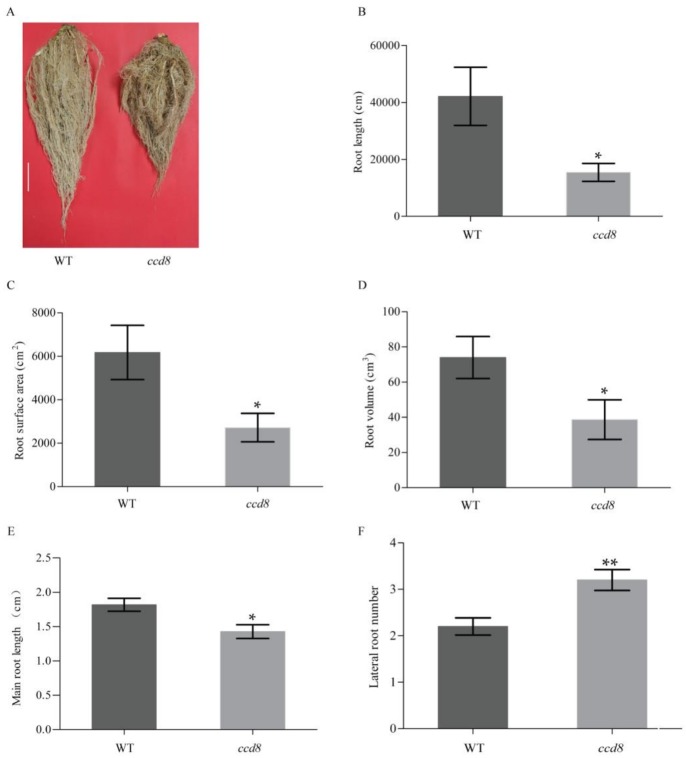
Phenotypic characterization of *ntccd8* mutant plants roots. (**A**) Root morphology of wild-type and *ntccd8* mutant plants at 50 day after transplantation. The scale bars equal to 6 cm. Total root length (**B**), root surface area **c** and root volume (**D**) were measured in 50-day-old wild-type and *ntccd8* mutant plants. (**E**,**F**) Average main root length and average lateral root number in wild-type and *ntccd8* mutant 10-day-old seedlings. Values are the means ± SD (*n* = 20). The asterisks indicate statistically significant differences in comparison to wild-type plants (* *p* < 0.05 and ** *p* < 0.01; Student’s *t* test).

In addition to the aboveground phenotypes, the underground root morphology was also investigated in *ntccd8* plants. The root length, area and volume were measured and analyzed in 50-day-old plants grown in pots. The mutant plants had a reduction in root mass and a smaller root system compared to wild-type plants (Figure 5A). The total root length, area and volume of *ntccd8* mutants were 2.7, 2.2, and 1.9 times lower than that of wild-type plants, respectively (Figure 5B–D). To test whether *ntccd8* mutations affects root architecture of seedlings, the primary root length and lateral root number were measured in seedlings. The *ntccd8* mutants had a significantly reduced primary root that was 44% shorter than wild-type seedlings (Figure 5E). However, the lateral root number in *ntccd8* mutants was higher than that of wild-type seedlings (Figure 5F).

**Figure 6:**
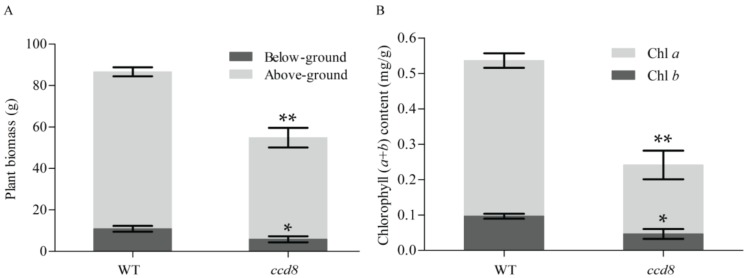
Plant biomass and chlorophyll content in wild-type and *ntccd8* mutant plants. (**A**) Dry weight of root and aerial tissue in 50-day-old wild-type and mutant plants. Data are the means ± SD (*n* = 3). (**B**) Quantification of total chlorophyll (Chl *a* and Chl *b*) in four-month-old plants leaves (*n* = 3 for wild-type and mutant plants). The asterisks indicate statistically significant differences compared to wild-type plants (* *p* < 0.05 and ** *p* < 0.01; Student’s *t*-test).

To investigate whether targeted *ntccd8* mutations affects plant biomass, the dry weight of leaves, stems and roots of *ntccd8* mutants and wild-type plants were determined after 50 days of growth. The mutants had a significant decrease in dry weight compared to wild-type plants (Figure 6A). However, the root-to-shoot ratio was similar between both plant genotypes (Figure S4).

### 2.6. Targeted NtCCD8 Genes Mutations Affects Plant Senescence

Leaf color changes from green to yellow after chlorophyll degradation. The leaves and stems of four-month-old *ntccd8* mutants were a pale green color or turned dark grey, suggesting a faster senescent process in *ntccd8* mutants than in wild-type plants (Figure S6). The amount of chlorophyll in leaves collected from *ntccd8* mutants was measured and analyzed, chlorophyll a and b (Chl *a* and Chl *b*) levels in the leaves of *ntccd8* mutants were significantly lower than in wild-type plants, which had twice as much in total chlorophyll content that the mutants (Figure 6B).

## 3. Discussion

The SLs signaling system has been well studied in *Arabidopsis*, pea and rice [39,63,64]. To our knowledge, this study is the first to report SL biosynthetic genes in tobacco. Common tobacco (*N. tabacum*) is an allotetraploid (2*n* = 48 resulting from both parents chromosomes sets being present in the gametes) that resulted from a *Nicotiana sylvestris* (2*n* = 24) and *Nicotiana tomentosiformis* (2*n* = 24) hybridization [69,70]. The two closely related genes that were cloned and characterized in the present study, *NtCCD8A* and *NtCCD8B*, encode the tobacco carotenoid cleavage dioxygenase NtCCD8A and NtCCD8B, respectively. When their amino acid sequences were aligned with those of *N. sylvestris* (NsyCCD8) and *N. tomentosiformis* (NtomCCD8), respectively, results showed that NtCCD8A originated from NtomCCD8 and NtCCD8B originated from NsyCCD8 (Table S1). NtCCD8A and NtCCD8B protein sequences were more similar to PhCCD8, and the phylogenetic tree revealed these three proteins were within the CCD8 cluster. The result indicates that the function of CCD8 is conserved in plant species. Although *NtCCD8A* and *NtCCD8B* were expressed in all tissues, they were most abundant in the roots (Figure 2A). Their expression pattern is consistent with the *CCD8* expression in *Arabidopsis*, petunia, pea, tomato and potato [46,49,50,58,71,72]. However, a different CCD8 expression pattern was observed in rice and chrysanthemum, the expression of *CCD8* in these species was predominantly in stem tissues [22,57]. These differences suggest that SLs can contribute to the regulation of shoot and root growth in a species-specific manner. 

*NtCCD8* genes play an important role in regulating plant architecture, which is an important trait that can be defined by the number of lateral branches and their length. Shoot branching is a highly plastic developmental process regulated by hormones, developmental stage and environmental factors [16,73,74,75]. The three hormones auxin, SLs and cytokinin are involved in shoot branching [17,76,77,78,79]. Recently, the mechanisms of SLs in the regulation of shoot branching have become the focus. The number of branches and leaves in *ntccd8* mutants was higher than that in wild-type plants (Figure 4D,F). In *ntccd8* mutants, axillary buds were already visible at the leaf axils during the early vegetative stage and subsequently grew into lateral branches (Figure S7). During later growth stages, some of the lateral branches in *ntccd8* mutants had the same length as the main stem (Figure S7B). This branching phenotype has been observed in SL biosynthesis and signaling mutants of several plant species, including *Arabidopsis*, petunia, pea, rice and maize [45,49,55,80,81]. In addition, the reduction of *CCD7* or *CCD8* expression in tomato, poplar, potato, kiwifruit and *Lotus japonicus* have been shown to increase stem branching [43,46,54,56,58,82]. Research has shown that CCD7 and CCD8 proteins are required for the production of SLs, which are thought to be involved in the inhibition of branch development [12,14]. SLs were not detected in the root extracts of *ntccd8* mutants (Figure S2). Thus, the branching phenotype observed in *ntccd8* mutants appears to be the result of SL loss. The *ntccd8* mutant plants had decreased main stem length compared to wild-type plants because of a reduced internode length (Figure 4E), which is in agreement with that found in petunia, *Lotus japonicus* and maize [49,55,82]. The reduction observed in reduced plant height of in *ntccd8* mutants might be because of the due to nutrients being partly used for increased lateral branch growth.

The interaction between auxin, SLs, and cytokinin has been demonstrated to synthetically regulate shoot branching in several plant species [32,34,78]. Auxin can up-regulate the expression levels of *CCD7* and *CCD8*, as has been described for the gene homologs *RMS1* in pea, *MAX4* in *Arabidopsis*, *D10* in rice and *CCD8* in chrysanthemum [22,57,78,83]. In the present study, the application of exogenous auxin increased *NtCCD8A* expression in tobacco (Figure 2C). However, *NtCCD8B* expression decreased when auxin was applied (Figure 2C). This differences between *NtCCD8A* and *NtCCD8B* expression might be due to a negative feedback regulation mechanism in SLs biosynthesis, which has been characterized in rice, petunia and *Arabidopsis* [22,49,84]. For example, the expression of *MAX4* in *Arabidopsis* and that of *CCD8* in chrysanthemum were inhibited by the application of the SLs analog GR24 [57,84]. Thus, the exogenous application of auxin might have resulted in the up-regulation of *NtCCD8A* expression, which lead to the SLs production and the accumulation of SLs that then inhibited the expression of the *NtCCD8B* gene. Hence, *NtCCD8A* would be the first response gene in SL biosynthetic regulation, which is consistent with its higher expression in root tissues compared to *NtCCD8B*. ABA is an important phytohormone that regulates adaptive responses to abiotic stress and is involved in many physiological processes in plants [85]. Recently, the role of SLs in plant stress response has been reported to play a positive role in the regulation of stress networks [31,86,87,88]. In *Arabidopsis*, *MAX3* and *MAX4* expression was induced by high salinity and ABA treatments [31]. The expression of the SL-biosynthesis gene *SlCCD7* and SLs production in tomato were induced by drought [89]. In tobacco, the application of exogenous ABA led to a three-fold increase in *NtCCD8B* transcript levels after 6 h of treatment, whereas *NtCCD8A* transcript levels were maintained (Figure 2C). This suggests that *NtCCD8B* upregulated in response to stress, which leads to an increase in SLs production that activates the ABA-dependent signaling networks necessary for the plant to adapt to various conditions.

In addition to regulating shoot architecture, SLs have also been shown to affect different aspects of root development [90]. It has been reported that maize and rice mutants have shorter primary roots than wild-type plants [44,55]. In *Arabidopsis*, *max3* and *max4* mutants have more lateral roots than the wild-type plants [25]. In *Medicago truncatula*, lateral root number was significantly reduced when plants were treated with the synthetic strigolactone analogue GR24 [91]. Results of the present study also demonstrate SLs regulatory functions in root architecture, all *ntccd8* mutant plants had a significant reduction in root length and a significant increase in lateral root number compared to wild-type plants (Figure 5E,F). Previous findings suggested that longer primary roots result from increased cell elongation [92]. A small root system phenotype was observed in *ntccd8* mutants, and their total root length, surface area and volume were lower than wild-type plants (Figure 2). This root phenotype is consistent with that of *zmccd8* mutants [55]. However, in *Lotus japonicus*, *LiCCD7*-silenced plants had greater total root length, area and volume than wild-type plants [82]. These results indicate that the effect of SLs on the regulation of root architecture depends on the plant species. Finally, *ntccd8* mutant plants had reduced above- and below-ground biomass compared to that of wild-type plants, but their root-to-shoot biomass ratios were similar (Figure 6A and Figure S4). This observation is consistent with that made in the petunia *ccd8*/*dad1* and *ccd7*/*dad3* mutants, but these exhibited an increased shoot-to-root ratio compared to wild-type plants [45,49]. The root biomass of *LiCCD7*-silenced plants was higher than that in wild-type plants [82]. These differences might be because of differences in species physiology and growth conditions.

Accelerated leaf senescence and decreased total chlorophyll (Chl *a+b*) content were observed in ntccd8 mutants compared to wild-type plants (Figure 6B and Figure S6). The timing of leaf senescence can be determined by reproductive development [82]. Hence, the early flowering of *ntccd8* mutants also indicated accelerated senescence when compared to wild-type plants (Figure S5). This is in agreement with that observed in *StCCD8*-RNAi potato plants [58]. However, many studies reported a delayed senescence phenotypes in SLs biosynthesis mutants in petunia, *Arabidopsis* and *Lotus japonicus* [28,49,82]. In pea and tomato, SL mutants do not have a delayed leaf senescence phenotype [43,46,83]. These results suggest that the function of SLs during leaf senescence differs depending on the plant species, and could be related to differences in environmental cues and plant hormones.

In summary, to the best of our knowledge, this study is the first to report an SL-dependent phenotypes in tobacco. *NtCCD8* genes are required for SL biosynthesis in tobacco and play an important role in regulating plant shoot and root development and growth. Targeted *NtCCD8* mutations influence the vegetative developmental processes in tobacco, including plant height, internode length, branch number and primary root growth. In addition, the effects of SLs on the regulation of leaf senescence were also described. Further research is required to characterize the molecular mechanism of SL signaling pathways that control plant architecture, as well as the mechanisms of SL interactions with other plant hormones.

## 4. Materials and Methods

### 4.1. Plant Materials, Growth Conditions and Treatments

Seeds of tobacco (*Nicotiana tabacum* L. ‘Honghuadajinyuan’) and of the homozygous transgenic NtCCD8 knockout lines were surface-sterilized and grown on Murashige and Skoog (MS) Basal Medium (PhytoTechnology Laboratories*^®^*, Kansas, MO, USA) containing 30 g/L sucrose and 8 g/L agar. One-month-old seedlings were transferred to pots with a peat moss to perlite mixture ratio of 3:1 and kept in a greenhouse under long-day conditions (16 h light/8 h dark; 25 °C) or subject to natural season conditions.

For plant hormone treatments, one-month-old seedlings were placed in full-strength Hoagland solution containing final concentrations of 100 μM abscisic acid (ABA) and 1-Naphthaleneacetic acid (NAA) [93]. For phosphate starvation, one-month-old seedlings were grown in half-strength Hoagland solution without phosphate for one week. After both treatments, the whole seedlings treated with hormones and the roots of plants treated with phosphate starvation were sampled and immediately frozen in liquid nitrogen for RNA purification.

### 4.2. Cloning of Tobacco NtCCD8A and NtCCD8B Genes

Two NtCCD8-like translated nucleotide sequences were obtained from the Sol Genomics Network (SGN) *Nicotiana tabacum* BX genome database (http://solgenomics.net/organism/Nicotiana_tabacum/genome) using the tomato SlCCD8 and potato PhCCD8 protein sequences as query subjects in a basic local alignment search tool (tBLASTn; Available online: https://solgenomics.net/tools/blast/) with default settings. Two full-length *NtCCD8* coding sequences were amplified from tobacco root tissue cDNA by PCR, using specific primers (Table S3) and the Phusion*^®^* high-fidelity DNA polymerase (NEB Inc., Ipswich, MA, USA). Successfully amplified PCR products were cloned into the pEASY-Blunt Zero Cloning Vector (TransGene, Beijing, China) and sequenced.

### 4.3. Phylogenetic Analysis

Multiple protein sequence alignments of CCD8 orthologs from *Arabidopsis*, tomato, petunia and tobacco were performed in Clustal X version 1.8 using default parameters (European Bioinformatics Institute, Hinxton, UK). To construct the phylogenetic tree, CCD8, CCD7, CCD4 and CCD1 protein sequences from several plants were aligned. An unrooted phylogenetic tree based on the CCD proteins alignment was constructed in MEGA Version 4.0 (Arizona State University, Tempe, AZ, USA), using the Neighbor-Joining (NJ) method with parameters of pairwise gap deletion and 1000 bootstraps.

### 4.4. RNA Extraction and Gene Expression Analysis

To detect the expression patterns of *NtCCD8A* and *NtCCD8B* in tobacco, root, stem, leaf and flower tissues of 70-day-old tobacco plants were collected, immediately frozen in liquid nitrogen, and kept at −80 °C until RNA extraction.

Total RNA from plant tissues was isolated with the RNeasy*^®^* Plant Mini Kit and treated with the RNase-free DNase kit (Qiagen, Duesseldorf, Germany) according to the manufacture’s protocols. Total RNA concentration was quantified on a NanoDrop ND-1000 Spectrophotometer (Thermo Fisher Scientific, Waltham, MA, USA). Total RNA (1 μg) was used for first-strand cDNA synthesis using M-MLV Reverse Transcriptase (Promega, Madison, WI, USA). Quantitative reverse transcriptase PCR (qRT-PCR) was performed using SYBR Premix-Ex Taq (TaKaRa, Bio, Inc., Otsu, Japan) and gene specific primers (Table S3). The tobacco elongation factor-1α (EF1α) gene was used as an internal control for gene expression analysis. A minimum of three biological and technical replicates was conducted for each analysis. The 2^−ΔΔ*C*t^ method was used to calculate the gene expression [94].

### 4.5. Plasmid Construction

A Cas9/gRNA vector was constructed as previously described with some modifications [65]. A BsaI restriction site in the Cas9 coding sequence was removed by synonymous mutation and the BbsI restriction site in the guide RNA (gRNA) expression cassette was replaced with BsaI, The cassette was digested with NotI and EcoRI restriction enzymes and then inserted into the pORE-Cas9 binary vector. For targeted *NtCCD8* modifications, two 24-bp DNA specific target oligonucleotides were synthesized (Table S3). These oligos were phosphorylated and annealed, and then inserted into the BsaI site of the pORE-Cas9 binary vector.

### 4.6. Plant Transformation and Mutant Analysis

The pORE-Cas9 binary vectors containing the gRNA and Cas9 expression cassettes were transformed into *Agrobacterium tumefaciens* LBA4404 by the freeze-thaw method. The positive clons were then used to produce *NtCCD8* mutant tobacco plants with the leaf discs method [95]. Kanamycin-resistant seedlings were obtained and mutants were identified. DNA was extracted from T_0_ transgenic lines using the DNeasy*^®^* Plant Mini Kit (Qiagen, Hilden, Germany). To detect mutations, the genomic regions including the Cas9/gRNA target sites were amplified by PCR using the specific primers (Table S3). The PCR products were directly sequenced using Sanger sequencing, and the resulting reads were aligned with wild-type sequences to detect candidate mutant lines. To investigate the inheritance of CRISPR/Cas9-induced targeted *NtCCD8* modifications in later generations, three T_0_ lines with biallelic mutations were self-pollinated and their T_1_ descendants were transferred to soil and grown to maturity. Mutant Cas9/gRNA-free tobacco lines were genetically identified using the specific primers (Table S3). T_2_ plants were then used for further analysis.

### 4.7. Phenotypic Analysis

Plant height, leaf number, internode length and stem perimeter were measured in 20-day-old, 40-day-old, 50-day-old, 60-day-old and 90-day-old wild-type and target knockout plants. Plant height was defined as the plant length from the soil level to the the highest leaf. Nodes and internodes were numbered from the first leaf node at the bottom of the plant to its upper parts, and internode length was calculated as the ratio of plant height to internode numbers. Stem perimeter was measured at the third internode counting from the apex. Shoot branching was monitored and analyzed based on the number and length of branches longer than 0.5 cm. The number and length of shoot branching were also counted and measured. The dry weight of above- and below-ground biomass was also measured. All the above-mentioned traits were described at least six plants from each independent line. The roots of tobacco mutants were collected from three individual plants and imaged with a scanner. The total root length, surface area and volume were then analyzed using the WinRhizo*^®^* image analysis system (Regent Instruments Inc., Québec city, QC, Canada). For the analysis of seedling root growth, 7-day-old seedlings of wild-type and mutant plants were grown in small square Petri dishes (12 cm × 12 cm) on solid MS medium. After ten days, the phenotype of seedlings was photographed, and the number of lateral roots was counted manually and the main root lengths were measured using ImageJ 1.49 software (http://rsb.info.nih.gov/ij/index.html).

### 4.8. Sls Extraction and Analysis

For SLs analysis, roots from mutant and wild-type plants were harvested 50 day after transplanting, frozen in liquid nitrogen and freeze-dried. The procedure of SL extraction was performed as previously described [96]. Freshly frozen root samples (0.5 g) were ground in a mortar using liquid nitrogen and extracted with 2 mL of ethyl acetate in 5 mL microcentrifuge tubes. Samples were vortexed and sonicated for 15 min in an Elmasonic S150 bath (Elma Ultrasonic, Singen, Germany), and then were centrifuged for 10 min at 2500× *g*. The supernatant was gently transferred to another set of 5 mL microcentrifuge tubes and the pellets was re-extracted with another 2 mL of ethyl acetate. The supernatants were combined and the ethyl acetate was evaporated to dryness under vacuum conditions. The dried fractions were re-suspended in 150 μL of acetonitrile: water (25:75, *v*/*v*) solution, filtered, and subjected to ultra-performance liquid chromatography tandem spectrometry (UPLC-MS/MS) analysis.

SLs analysis were carried out using the method as previously reported with minor modifications [96]. A quadrupole Orbitrap hybrid mass spectrometer coupled to UltiMate3000 UPLC system (Thermo Fisher Scientific) was used for SL detection. Chromatographic separation was achieved with a ZORBAX BS-C18 (2.1 × 100 mm 1.8 μm, Agilent (Agilent Technologies Inc., Santa Clara, CA, USA)). Gradient elution was performed in a mobile phase A (0.1% formic acid in water) and B (0.1% formic acid in acetonitrile) at 0.4 mL min^−1^ flow rate and at 50 °C. Mobile phase’s initial composition (5% B) was maintained for 2 min, increased from 5 to 50% B in 8 min, from 50 to 90% in 1 min, held at 90% for 0.1 min, and returned to the initial conditions using a 0.2-min gradient. Finally, the column was equilibrated for 2.8 min before the next injection start. Injection volume was 15 μL. The mass spectrometer was operated in positive electrospray ionization mode (ESI+). The spectra were recorded using target-sim mode (331.153 *m*/*z*) and full scan mode, covering a mass range from 100 to 1000 *m*/*z*. The resolution was set at 70,000. 

Parameters of for the ion source were set as follows: the mass spectrum (MS) spray voltages 3.5 kV, capillary temperature 320 °C, probe temperature 100 °C, sheath gas pressure 35 arb, auxiliary air pressure 5 arb. Date acquisition and analysis were performed using the Thermo Xcalibur software (Thermo Fisher Scientific).

### 4.9. Chlorophyll Analysis

To extract chlorophyll, leaves located at identical positions along the main stem were sampled from three wild type and three mutant plants, and analyzed as described previously [97]. Leaves were cut into small pieces, weighted, and transferred into a 10-mL centrifuge tube containing 5 mL dimethylsulfoxide (DMSO). Chlorophyll extraction was performed for 24 h in the dark at room temperature. The supernatant extracts were then placed in a spectrophotometric cuvette and chlorophyll content was calculated based on the absorbances at 649 and 665 nm wavelengths, which were measured in three technical replicates from each biological replicates.

### 4.10. Off-Target Analysis

The potential off-target effects resulting from *NtCCD8* genes editing were analyzed and evaluated as previously reported [65].

### 4.11. Statistical Analysis

Statistical analysis was performed on all data using Student’s *t*-test. GraphPad Prism 5.0 (GraphPad Software, La Jolla, CA, USA) was used for plotting data and imaging.

## Figures and Tables

**Table 1:** Percentage of transgenic T_0_ plants with mutations in *NtCCD8* genes produced using the CRISPR/Cas9 system.

Mutation Gene Target	Mutation Rate (%)	Number of Plants Examined	Number of Plants with Mutation
*NtCCD8A*	33.3%		8
*NtCCD8B*	27.8%	18	5
*NtCCD8A* and *NtCCD8B*	16.7%		3
Total	77.8%	18	14

**Table 2:** Segregation patterns of CRISPR/Cas9-induced mutations in the *NtCCD8* genes during the T_0_ to T_1_ generations.

Line ^a^	Target Genes	T_0_		T_1_	
		Zygosity ^b^	Genotype ^c^	Segregation Ratio	Cas9 ^d^
T_0_-3	*NtCCD8A*	Bi-allele	i1,d2	5i1:10i1d2:5d2	19+: 1−
	*NtCCD8B*	Bi-allele	d1,d3	5d1:12d1d3:3d3	
T_0_-5	*NtCCD8A*	Bi-allele	i1,d2	6i1:10i1d2:4d2	16+: 4−
	*NtCCD8B*	Bi-allele	d1,d2	5d1:6d1d2:9d2	
T_0_-14	*NtCCD8A*	Bi-allele	i1,d2	3i1:12i1d2:5d2	18+: 2−
	*NtCCD8B*	Bi-allele	i1a,i1b	20i1	

^a^ Line name is in the format of T_0_-# with indicating the T_0_ generation, # indicating the plant #. ^b^ The zygosity of bi-allele in T_0_ plants is putative. ^c^ The genotype of individual plants according to the sequencing results. ^d^ +, the number of Cas9 sequences that were detected; −, the number of Cas9 sequences that were not detected. Mutation types: d#x and i#x; d and i indicate nucleotide deletions and insertions, respectively. ‘#’ represents the number of nucleotide deletions or insertions. ‘x’ is used to show the different mutations at the same target site. For example, i1a and i1b represent the insertion of one different nucleotide at the same site.

## Supplementary Materials

Supplementary materials can be found at http://www.mdpi.com/1422-0067/19/4/1062/s1.
