# Supplementary material for: CRISPR/Cas9-Mediated Mutagenesis of Carotenoid Cleavage Dioxygenase 8 (CCD8) in Tobacco Affects Shoot and Root Architecture
Source: Int J Mol Sci. 2018 Apr 2;19(4):1062. doi: 10.3390/ijms19041062 (PMC5979566; doi:10.3390/ijms19041062)
Supplement: Supplementary file 1 [file ijms-19-01062-s001.pdf]

Supplementary Materials

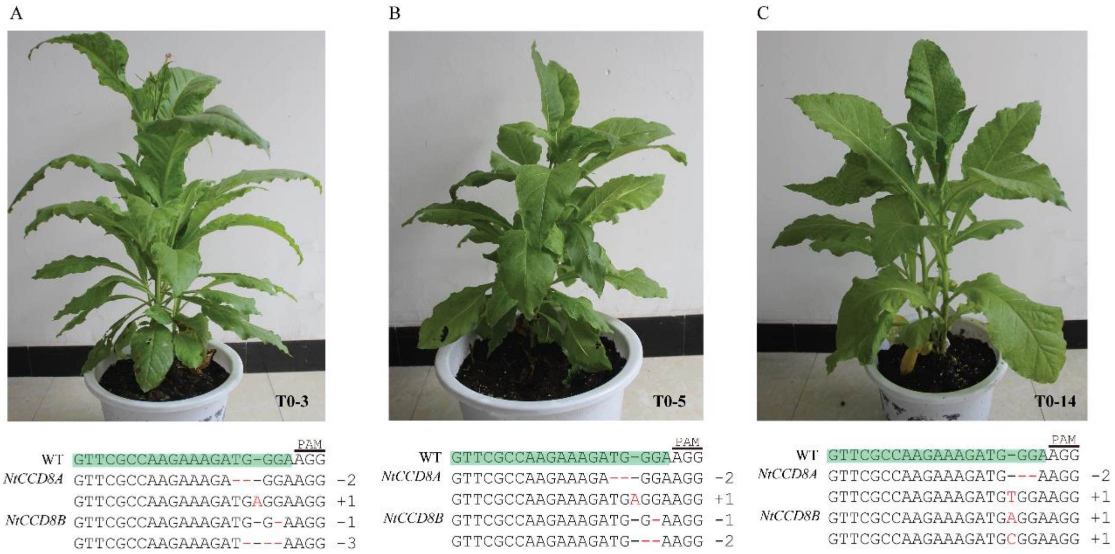

Supplementary Figure S1. Phenotypes of *ntccd8* mutant T_0 plants. Three bi-allelice mutant plants (A, B, C) were obtained showing distinct phenotypes. Sequencing results of these mutant plants (A, B, C) were aligned with the reference genome sequence. Indels are shown in red nucleotides and dashes. The numbers on the right side indicate the size of the indels.

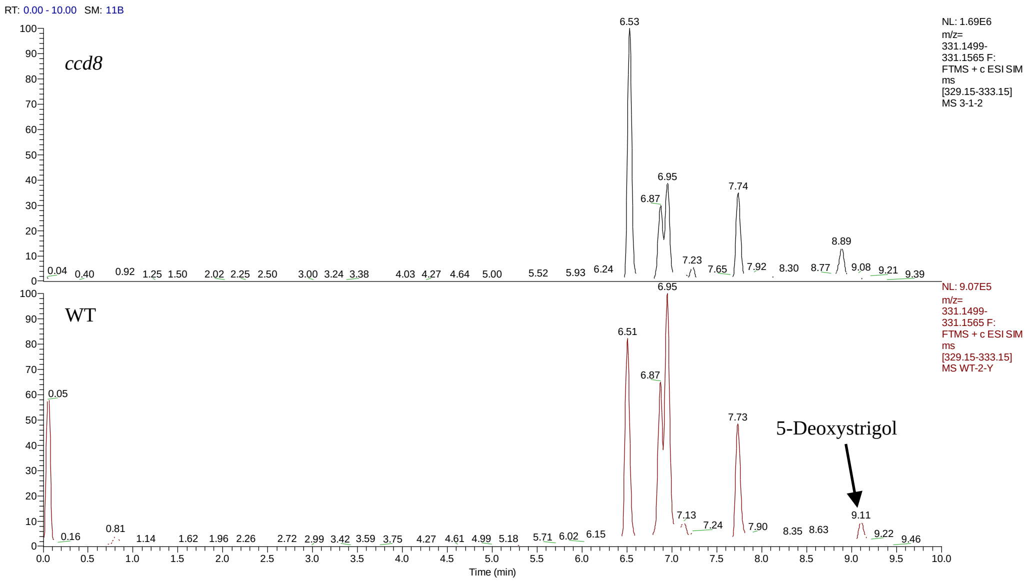

Supplementary Figure S2. Strigolactone analysis in tobacco root extracts. UPLC-MS/MS was used to detecte the SLs extracted from *ntccd8* mutants (up) and wild-type (down) plants roots at 50 d after transplanting. No significant peaks were seen in the *ntccd8* mutant.

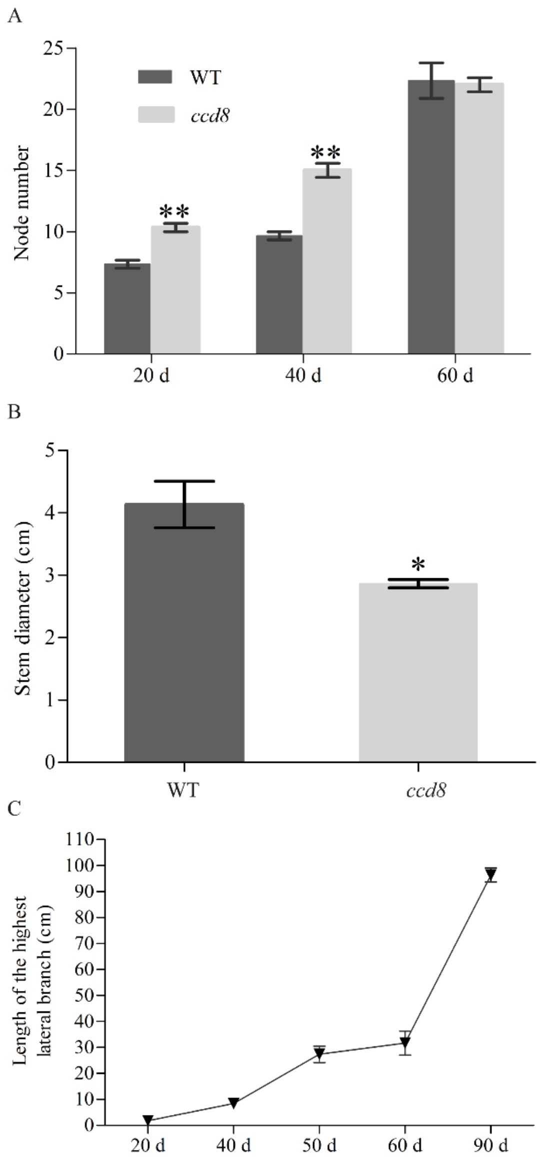

Supplementary Figure S3. Node number, stem diameter and length of the highest branch in *ntccd8* mutant and wild-type plants. Main stem length (A) was determined at 20 d, 40 d and 60 d after transplanting. Stem diameter (B) was measured at the third internode from the apex in 50-day-old plants. (C) Length of the highest branch of *ntccd8* mutant plants at 20 d, 40 d, 50 d, 60 d and 90 d after transplanting. Data are the means ± SD (n>9). The asterisks indicate statistically significant differences in comparison to wild-type plants ($^{*}P < 0.05$ and $^{**}P < 0.01$; Student's *t*-test).

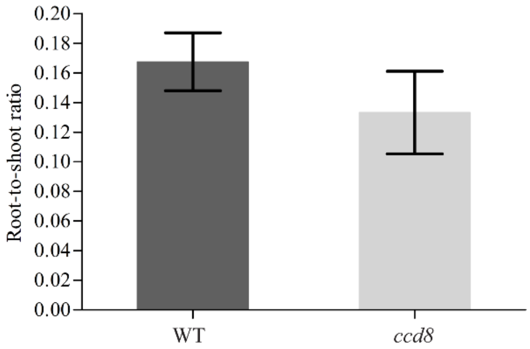

Supplementary Figure S4. Root-to-shoot biomass ratio in *ntccd8* mutant and wild-type plants.

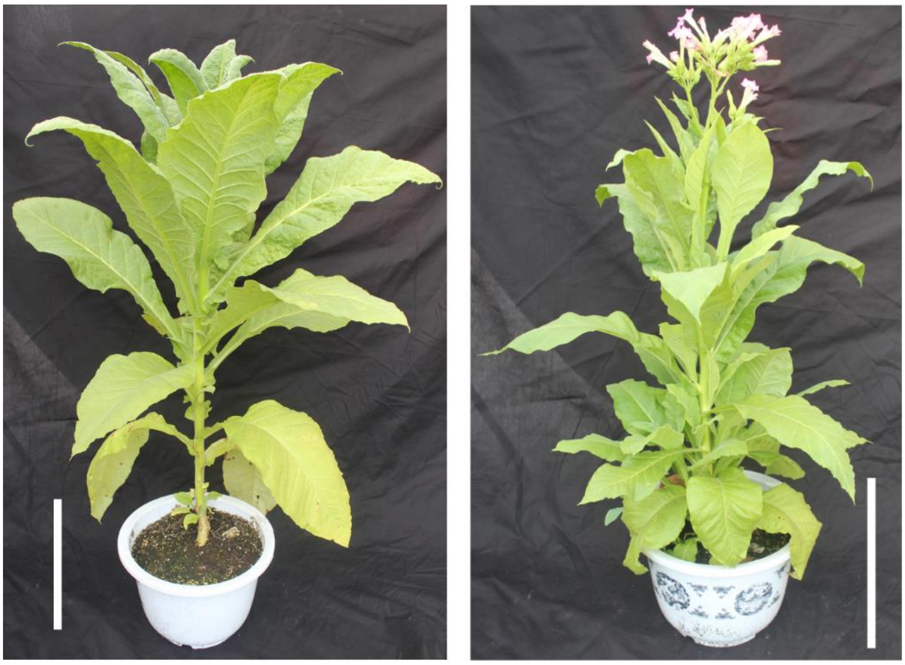

Supplementary Figure S5. Regulation of flowering time in *ntccd8* mutant plants. The *ntccd8* mutants showed an earlier flowering than wild-type plants at 60 d after transplanting. The scale bars equal to 25 cm.

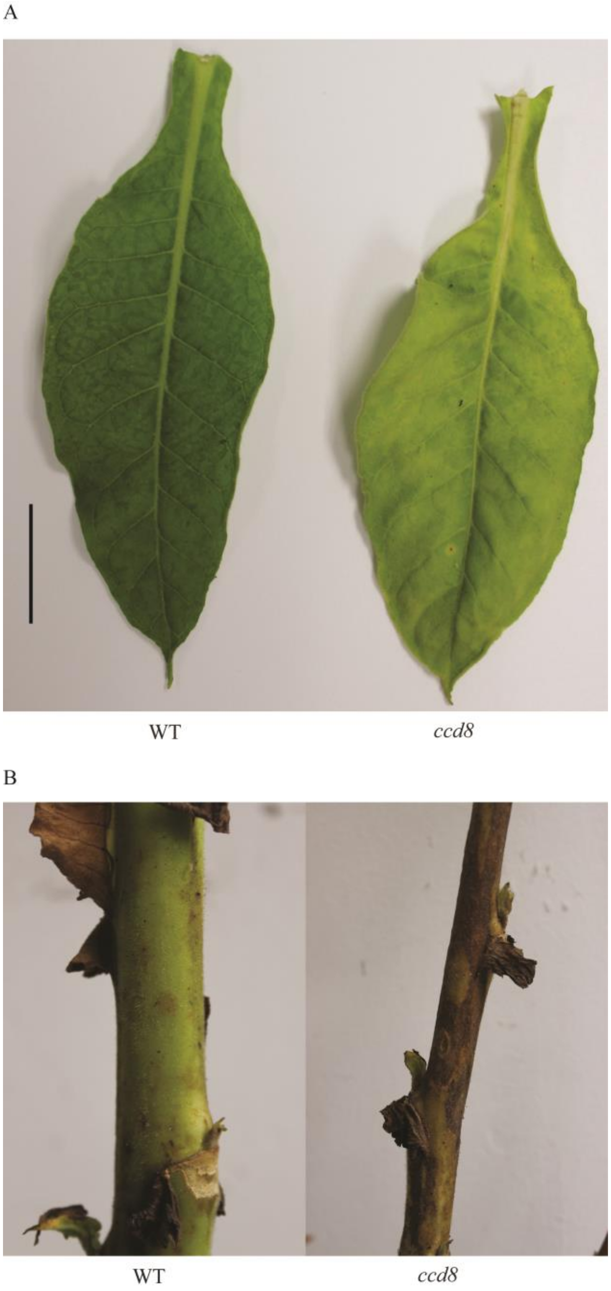

Supplementary Figure S6. Regulation of the senescence process in *ntccd8* mutants. Representative images of the leaf (A) and stem (B) appearance in four-months-old wild-type and *ntccd8* mutant plans. The scale bars equal to 2 cm.

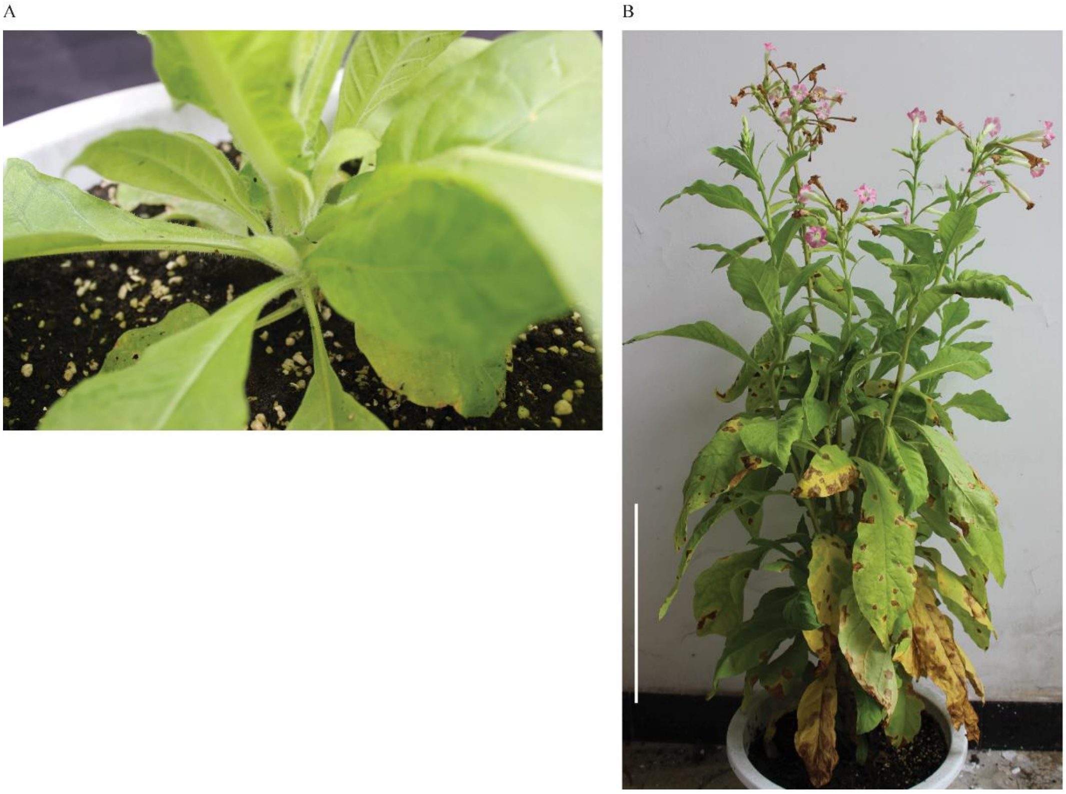

Supplementary Figure S7. Phenotypes of the *ntccd8* mutants during early and late growth stages. Representative image of the growth of mutant plants at 20 d (A) and 90 d (B) after transplanting. The scale bars equal to 25 cm.

Supplementary Table S1. Comparison of the deduced amino acid sequences of CCD8 proteins in various species. Numbers indicate % identity between the predicted proteins.

	NtCCD8A	NtCCD8B	SlCCD8	PhCCD8	AtCCD8	NsyCCD8	NtomCCD8
NtCCD8A							
NtCCD8B	96%						
SlCCD8	88%	88%					
PhCCD8	90%	92%	87%				
AtCCD8	68%	76%	66%	68%			
NsyCCD8	96%	100%	88%	92%	76%		
NtomCCD8	100%	96%	88%	90%	68%	96%	

Nt, *Nicotiana tabacum*; At, *Arabidopsis thaliana*; Ph, *Petunia hybrida*; Sl, *Solanum lycopersicum*; Nsy, *Nicotiana sylvestris*; Ntom, *Nicotiana tomentosiformis*.

Supplementary Table S2. Examination of mutations in the putative CRISPR/Cas9 off-target sites.

Target genes	Name of putative off-target site	Sequence of the putative off-target site	No. of mismatching bases	No. of plants sequenced	No. of plants with motations
NtCCD8A and *NtCCD8B*	off1	GGTTCCAAAGAAAGATGGGAAGG	4	12	0

The PAM moti (NGG) and mismatching bases are shown in red and green letters, respectively.

Supplementary Table S3. List of primers used in this study.

Primer name	Sequence (5'-3')	Usage
NtCCD8A-F	ATGGCTTCTTTTGCTTCTTCAT	The amplication of complete
NtCCD8A-R	CTATTTCTTTGGAACCCAACAA	*NtCCD8A* coding sequence
NtCCD8B-F	ATGGCTTCTTTTGCTTCTTC	The amplication of complete
NtCCD8B-R	CTATTTCTTTGCAACCCAGC	*NtCCD8B* coding sequence
qNtCCD8A-F	ACAGGCAAATCGAATCGGAG	For quantification of the relative
qNtCCD8A-R	GTGTTCGCGTTATCGGTTAGG	expression of *NtCCD8A*
qNtCCD8B-F	ACAGGCAAATCGAATCAGACG	For quantification of the relative
qNtCCD8B-R	GTGTTGGCGTTATCGGTTAGAG	expression of *NtCCD8B*
NtEF-F	GCATTGCTTGCTTTCACCCTT	The internal reference control
NtEF-R	AACCTCCTTCACGATTTCATCATACC	perimers for qRT-PCR
NtCCD8s-F	GATTGTTCGCCAAGAAAGATGGGA	For constructing gRNA of
NtCCD8s-R	AAACTCCCATCTTTCTTGGCGAAC	*NtCCD8s* target

F1	TTACTGTAACAAGATCCTTTCTGAC	PCR *NtCCD8A* target
R1	TTCATACTGTTCAGAAACCTTTGT	region
F2	TCTATTGTACCAATATCCTTTCTCA	PCR *NtCCD8B* target
R2	TGTCTGAATAAGTACTGAAACCTTT	region
Cas9-F	ATTTTCACCATTTACGAACG	PCR *Cas9* fragment
Cas9-R	AATTATTACATGCTTAACGTAATTC	
Off1-F	AAACTACCTACCGACCCTATTTGT	Detection potential
Off1-R	TAATAAAGGTTGTCTTTTCAGCATC	off-target mutations
